# Effectiveness and cost-effectiveness of an online transdiagnostic positive psychology intervention for patients awaiting mental health treatment and their loved ones: study protocol

**DOI:** 10.1186/s40359-025-03860-0

**Published:** 2025-12-18

**Authors:** Janôt Zinzen, Susan van Hooren, Ruben M.W.A. Drost, Xynthia Kavelaars, Pieter J. Rohrbach, Viviane Thewissen, Silvia M.A.A. Evers, Jantine J.L.M. Boselie

**Affiliations:** 1 Department of Clinical Psychology, Faculty of Psychology, Open University, P.O. Box 2960, Heerlen, 6401 DL The Netherlands; 2 Research Centre on arts therapies and psychomotricity, Zuyd University of applied sciences, P.O. Box 550, Heerlen, 6400 AN The Netherlands; 3 Department of Health Services Research, Care and Public Health Research Institute (CAPHRI), Faculty of Health, Medicine and Life Sciences (FHML), Maastricht University, P.O. Box 616, Maastricht, 6200 MD The Netherlands; 4 Department of Methodology & Statistics, Section Methodology and Statistics, Open University, P.O. Box 2960, Heerlen, 6401 DL The Netherlands; 5 Centre for Economic evaluation, National Institute of Mental Health and Addiction, Trimbos Institute, P.O. Box 725, Utrecht, 3500 AS The Netherlands

**Keywords:** Budget impact analysis, Economic evaluation, EHealth, Mental health treatment, Positive psychology intervention, Randomized controlled trial, Replicated single case design, Transdiagnostic, Transdiagnostic

## Abstract

**Background:**

Mental healthcare is under pressure, resulting in prolonged waiting lists for treatment. This is problematic, as time spent on a waiting list is associated with suboptimal recovery, more treatment dropout, and increased societal costs. An online transdiagnostic positive psychology intervention (eHealth PPI) may increase resilience (i.e., the ability to adapt to challenging experiences) and well-being among patients. Offering the same intervention to loved ones could be a novel contribution to enhancing positive effects among patients and loved ones. A study protocol is presented aimed at examining the clinical effectiveness, cost-effectiveness and implementation procedures of an eHealth PPI for patients with various mental health complaints awaiting treatment and their loved ones.

**Methods:**

Using two studies, the current manuscript proposes a threefold evaluation of an eHealth PPI consisting of 9 modules, focusing on: [1] clinical effectiveness, [2] economic evaluation, and [3] implementation procedures. To evaluate the procedural implementation of the eHealth PPI from a user-perspective, a replicated single-case design (RSCD) will be performed in study 1, with measurements at baseline, after each module, and post-intervention. In study 2, a randomized controlled trial (RCT) will be used to examine the clinical effectiveness, cost-effectiveness and implementation procedures of the eHealth PPI, using three arms: [1] PPI during the waiting period for patients, [2] PPI during the waiting period for patients and loved ones, and [3] treatment as usual (TAU), i.e., the conventional waiting period for patients. Following the waiting period, patients will receive a mental health treatment as usual. Study 2 will employ measurements at baseline, post-intervention, after 3, 6, and 9 months, 1 year following PPI completion, and 1 year following treatment completion. A key outcome is positive mental health, consisting of emotional, social, and psychological wellbeing. Other outcomes include, for instance, resilience, psychological complaints, quality of life, societal costs, and adherence.

**Discussion:**

Introducing a transdiagnostic eHealth PPI to various patients awaiting mental health treatment and their loved ones may improve treatment outcomes. Enhancing resilience through an eHealth PPI may reduce healthcare costs and health-related societal costs, potentially making it a cost-effective strategy compared to TAU.

**Trial registration:**

NL-OMON57370. Registered in the Overview of Medical Research in the Netherlands on 17 March 2025.

**Supplementary Information:**

The online version contains supplementary material available at 10.1186/s40359-025-03860-0.

## Background

Mental healthcare systems are facing increased care demands amidst staff shortages, resulting in prolonged waiting times for mental health treatment [1]. To address this challenge, certain countries have established national benchmarks to improve waiting times in mental healthcare, including Denmark, Finland, Ireland, Lithuania, New Zealand, Norway, Spain, United Kingdom, and The Netherlands [2]. Notably, up to 75% of patients with common mental health conditions are currently being treated within 6 weeks in the United Kingdom [2]. In The Netherlands, insurers and healthcare providers have agreed on a maximum acceptable waiting period of 14 weeks for most conditions (i.e., the Dutch Treeknorm) [3]. However, this norm is often not met in Dutch mental healthcare, as waiting times for patients exceed 14 weeks in approximately 30% of all cases [4]. This is problematic, as time spent on a waiting list is negatively associated with desirable treatment outcomes, including improvement and recovery [5]. In addition, it is associated with increased drop-out rates during treatment [6, 7] and increased costs for interim care provided by General Practice Mental Health Professionals (GP-MHP), which is used to bridge the waiting time [8]. While waiting, prospective patients may experience aggravating symptoms [9], compromising adequate recovery [10, 11]. In addition, aggravation of mental health symptoms is associated with decreased quality of life, delays in educational achievement [12], reduced productivity and increased absenteeism in the workspace [13, 14], causing a substantial burden of illness [15].

Apart from the costs within the healthcare sector, the societal costs of mental illness mostly manifest outside the healthcare sector [16]. Importantly, the annual excess cost per capita of having any common mental disorder is approximately €3,200 in The Netherlands [16]. Even though GP-MHP care may be delivered to some patients awaiting treatment, a structured treatment plan to bridge the waiting time is often not guaranteed [3], potentially causing patients to utilize other healthcare resources [15]. Although cost estimates of waiting times prior to receiving mental health treatment are not fully known on the population level, the waiting time itself is considered a predictor of mean economic costs for depression and anxiety disorders across all levels of clinical severity [17].

As waiting lists cannot be easily reduced in the short term, a potential alternative is to examine whether offering an online, resource-based intervention during the waiting period could improve existing treatment protocols for various patients awaiting mental health treatment. Resource-based interventions, including Positive Psychology Interventions (PPIs), aim to strengthen mental resources in human beings, including resilience [18]. In short, PPIs are empirically supported and aim to cultivate resilience by increasing positive factors (e.g., optimism, self-compassion) [19, 20]. In general, PPIs may help individuals cope with challenging situations and, in turn, increase wellbeing [18]. Enhancing protective factors may also reduce risk factors contributing to psychopathology [21]. Since GP-MHP care is not structurally available for all patients due to high demand during the waiting period, offering an evidence-based eHealth PPI may be an adequate alternative to reduce staff costs and to improve timely access to hybrid mental healthcare, potentially making it an effective and low-cost intervention.

The clinical effectiveness of PPIs has been demonstrated in patients with depression, anxiety and chronic pain [21–23]. Although it is currently unknown whether a transdiagnostic eHealth PPI could be effective in other mental disorders (e.g., autism spectrum disorder and personality disorders) due to a lack of prior studies, it is worth exploring whether a transdiagnostic eHealth PPI can be applied more broadly. There are no theoretical underpinnings for suboptimal PPI outcomes in other study populations. In contrast, PPIs were even able to produce meaningful clinical outcomes in a high-risk cohort of inpatients with suicidal complaints [24], highlighting their potential usefulness.

In most cases, the patient’s loved ones (e.g., partner, family, friends, colleagues) are not involved in treatment, even though it is well established that interpersonal connections, such as connections with loved ones, may further promote patient recovery [25]. Furthermore, research suggests that positive activities could promote positive relationships with others, which may mitigate interpersonal risk factors like loneliness [19]. Importantly, loved ones often become caregivers for patients, which may cause them to feel burdened. Consequently, loved ones may experience mental health complaints as well, requiring more demand for psychological care [26]. The effects of psychological interventions may be enhanced by involving the patient’s social network, as this may yield synergistic effects that strengthen an intervention’s overall impact [27]. Hence, PPIs targeting both patients and their loved ones may enhance patient recovery and yield positive effects among loved ones who may also experience mental health complaints.

While the clinical effectiveness of PPIs has been demonstrated systematically [21, 28–31], evidence on their cost-effectiveness — apart from two encouraging studies conducted in the general Dutch adult population by Schotanus-Dijkstra, Drossaert [32] and Bolier, Majo [33] — remains limited. In addition, the clinical effectiveness and cost-effectiveness of combining an eHealth PPI with a subsequent mental health treatment have generally not been evaluated, nor have these objectives been examined within a heterogeneous research population containing patients with various mental health conditions and their loved ones. To address these research gaps, an eHealth positive psychology intervention (eHealth PPI) will be developed, characterized by transdiagnostic treatment principles that can be applied broadly across mental health conditions. The proposed eHealth PPI will target transdiagnostic factors to support patients with various mental health conditions awaiting outpatient treatment and their loved ones. Addressing these gaps is highly relevant, especially in light of contemporary attempts to enhance the clinical value and financial sustainability of Dutch mental healthcare [34], such as the increasing focus on providing hybrid care [35]. Within hybrid mental healthcare, various eHealth applications are likely cost-effective, since they are generally considered cost saving for a range of mental disorders [36, 37]. In addition to assessing (cost-)effectiveness, evaluating procedural implementation is key for clinical practice. In order to avoid long term implementation barriers, digital mental health interventions should be financially and legally implementable within the organizational constraints of healthcare systems [38]. To optimize intervention success among participants, gathering data on its feasibility, acceptability, accessibility, adherence and engagement is imperative [39].

Considering the aforementioned research gaps, we aim to expand the scope of knowledge on eHealth PPIs and their transdiagnostic applications, and clinical and financial implications. To facilitate reproducibility and transparency of all scheduled methods in advance [40], a comprehensive study protocol was developed. In light of this purpose, the current paper presents a study protocol aimed at examining the (cost-)effectiveness and feasibility of an eHealth PPI for patients with various psychological complaints awaiting mental health treatment and for their loved ones.

## Objectives

Two experimental conditions will be established involving the eHealth PPI during the waiting period for [1] patients only and [2] for patients and their loved ones. Primarily, both experimental conditions will be compared to a [3] treatment as usual (TAU) condition, involving patients who will not receive the eHealth PPI during their waiting period. However, conditions 1 and 2 will also be compared to each other exploratively. In all three conditions, patients will receive a mental health treatment as usual after the waiting period. Using these three RCT conditions and a small pilot study (replicated single-case design), the study protocol will address objectives and hypotheses from three areas of expertise:

### Clinical effectiveness

#### Primary objective

Our primary clinical aim is to examine whether offering the eHealth PPI to various patients during the waiting period and their loved ones, followed by a mental health treatment for patients, is clinically effective in terms of improving positive mental health scores (primary outcome) at the 6-month and 12-month post-PPI measurement, compared to TAU. We hypothesize that introducing this eHealth PPI in condition 1 (PPI for patients only) and in condition 2 (PPI for patients and loved ones), followed by a mental health treatment, is clinically more effective, compared to condition 3 (TAU).

#### Secondary objective

Our secondary clinical aim is to evaluate whether offering the proposed eHealth PPI simultaneously to both patients and their loved ones during the waiting period (condition 2), followed by a mental health treatment for patients, is clinically superior to only involving patients in the eHealth PPI (condition 1). In addition, individual trajectories will be modelled to explore which patients are most likely to benefit from the eHealth PPI.

### Economic evaluation

#### Primary objective

Our primary economic aim is to examine whether delivering the eHealth PPI to patients during the waiting period and their loved ones, followed by a subsequent mental health treatment for patients, is more cost-effective from a societal perspective, compared to TAU. We hypothesize that offering this eHealth PPI to participants in conditions 1 and 2, followed by a mental health treatment, is cost-effective from a societal perspective, compared to condition 3 (TAU).

#### Secondary objective

Our secondary economic aim is to evaluate whether involving loved ones in the eHealth PPI during the waiting period, followed by a mental health treatment for patients, will yield additional cost-effectiveness benefits from a societal perspective, compared to only involving patients. We anticipate that involving loved ones in condition 2 of the eHealth PPI, followed by a mental health treatment for patients, will be cost-effective from a societal perspective, compared to condition 1.

### Implementation procedures

Our aim is to explore the acceptability, feasibility, engagement, adherence, and accessibility of the proposed eHealth PPI from a user-perspective.

## Methods

### Study designs

Two study designs will be developed. While study 1 will employ a replicated single case design (RSCD) to assess the preliminary intervention effects and procedural implementation of the proposed eHealth PPI, study 2 will conduct a randomized controlled trial (RCT) to examine the eHealth PPI more robustly in terms of clinical effectiveness, cost-effectiveness and procedural implementation.

#### Study 1: replicated single-case design

A pilot study employing a replicated single-case design (RSCD) will be conducted to examine the implementation procedures of the eHealth PPI from a user-perspective, focusing on: acceptability, feasibility, engagement, adherence, and accessibility. Following study enrollment, the RSCD will include ten participants (five patients and five loved ones) completing multiple measurements. These measurement phases include: pre-measurement, interim measurement after each intervention module, and a post-measurement. The dependent primary outcome variable to be measured is positive mental health, which consists of social, emotional, and psychological wellbeing [41]. Dependent secondary outcome variables include optimism, self-compassion, savoring, gratitude, resilience, and quality of life. Generally, an RSCD allows for the early identification of potential facilitators and barriers to intervention implementation, which may be used to inform adjustments to the intervention design and materials [42]. A total study time of 10 weeks is estimated for the participants.

#### Study 2: randomized controlled trial design

**Fig. 1:**
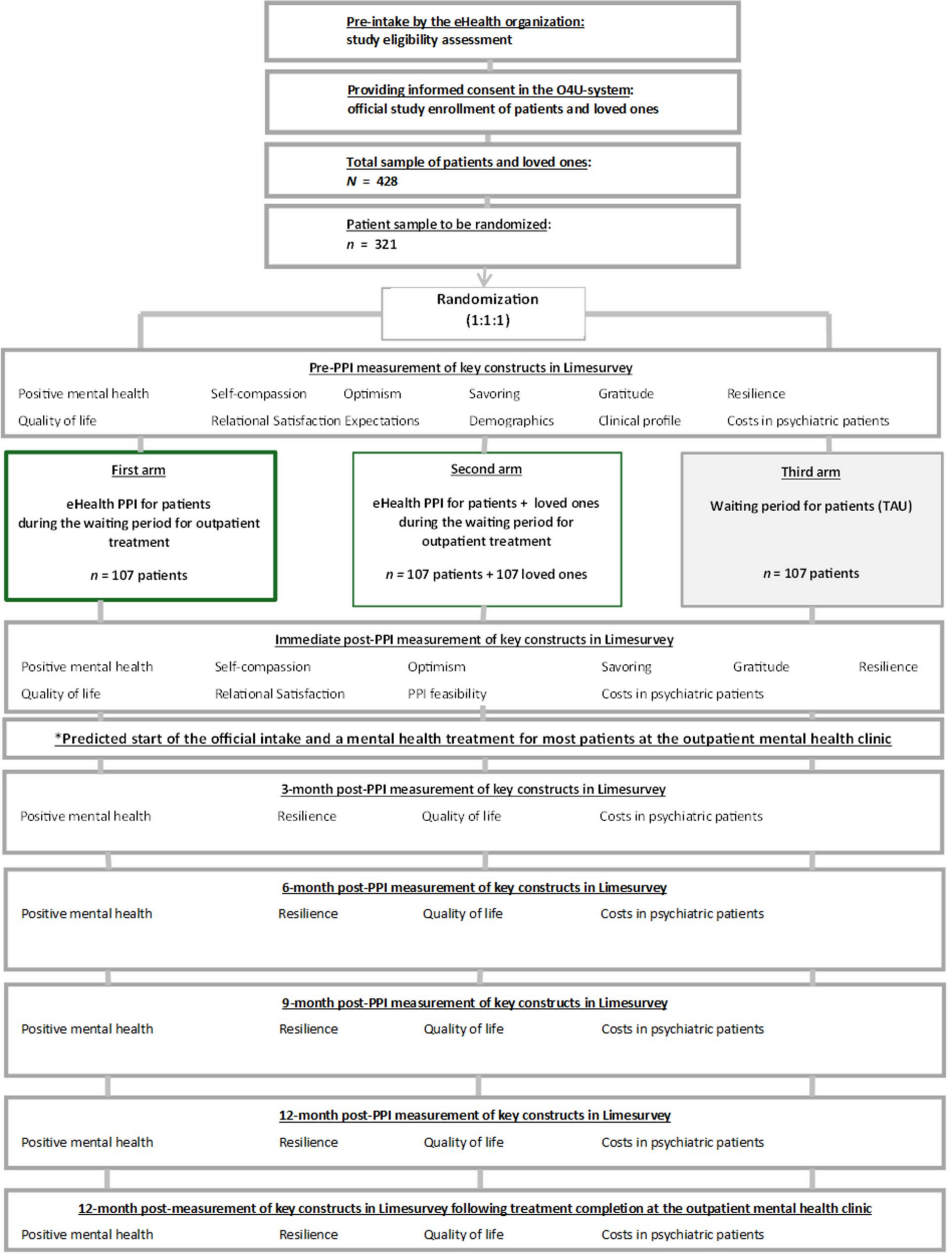
Flowchart of the proposed RCT (study 2). Note: *Predicted start of the official intake and a mental health treatment for most patients: Although the official intake and treatment could take place in principle after the pre-measurement, most patients will likely receive treatment more than 5 weeks after completion of the first post-measurement due to long waiting times in Dutch mental healthcare (i.e., > 14 weeks)

A three-arm RCT design will be implemented to examine the (cost-)effectiveness and procedural implementation of the eHealth PPI for patients awaiting mental health treatment without loved ones (arm 1) and in patients awaiting mental health treatment with their loved ones (arm 2), compared to patients not receiving the eHealth PPI during their waiting period for mental health treatment (TAU in arm 3). Following study enrollment, all participating patients will be assigned to one of the three RCT arms, using simple randomization with an equal 1:1:1 allocation ratio. A loved one will be added to each patient in arm 2. Participants will receive seven different measurements: a pre-PPI measurement, immediate post-PPI measurement, 3-month post-PPI measurement, 6-month post-PPI measurement, 9-month post-PPI measurement, 12-month post-PPI measurement and a final 12-month measurement following treatment completion at a Dutch mental health clinic. The dependent primary outcome variable will be positive mental health, which consists of social, emotional, and psychological wellbeing [41]. Dependent secondary outcome variables include optimism, self-compassion, savoring, gratitude, resilience, quality of life and costs in psychiatric patients. Independent background characteristics include demographics (e.g., age, sex, education), clinical profile (e.g., severity of complaints) and expectations. A flowchart of the RCT is presented in Fig. 1 on page 13 below, displaying the primary and secondary outcomes for each measurement.

### Study population

The study population will consist of Dutch patients facing a range of mental health symptoms and their loved ones. While the participating outpatient mental health clinic (i.e., Lionarons GGZ) does not provide treatment for acute suicidality and psychotic complaints, the most common clinical diagnoses of patients waiting for general or specialized mental healthcare at this clinic are depression, post-traumatic stress disorder, anxiety, personality disorders and developmental disabilities (e.g., autism spectrum disorder). The outpatient mental health clinic will be supported by Embloom, which is an eHealth organization identifying provisional diagnoses and care needs of incoming patients. In principle, loved ones are broadly defined as all individuals who are meaningfully connected to a patient [25]. This definition acknowledges the patient’s autonomy in deciding who qualifies as a meaningful connection (e.g., a formal partner, blood-related family member, close friend, or colleague). Hence, the current project may include a diverse range of valuable interactions between patients and their loved ones, which may increase the likelihood of patient inclusion. The anticipated research population will probably contain a broad spectrum of mental health diagnoses and vulnerable target groups.

Patients and their loved ones are eligible for both studies if they meet the following inclusion criteria during the recruitment procedure: all participants [1] are at least 18 years old, [2] are able to read and speak the Dutch language, and [3] have access to the internet. In addition, patients [4] have obtained an referral for treatment at the outpatient mental health clinic and [5] are able to appoint an adult loved one willing to participate. Patients will be excluded if they meet one of the following two criteria during the recruitment procedure: [1] the patient is referred back to their general practitioner by the eHealth organization supporting the outpatient mental health clinic, [2] the patient meets the criteria for severe acute suicidality or psychotic symptoms.

### Sample size

#### Study 1 (RSCD): sample size considerations

A small sample of ten participants will be included in study 1, comprising five patients and five loved ones, before scaling up to a fully powered trial. Using a replicated single-case design (RSCD), measuring a few single cases (*n* = 1) of patients and loved ones repeatedly is usually sufficient to examine relevant aspects of intervention implementation. In study 1, single cases will be followed over a prolonged period of time using repeated measurement phases prior to, during, and after the RSCD. Although the data obtained may have limited implications for group-level analysis, these measurement phases enable short-term detection of implementation experiences on the individual level, making the RSCD a relatively efficient design for small pilot studies [43, 44].

#### Study 2 (RCT): adaptive sample size design

An adaptive sample size design will be employed in study 2, estimating the required sample size prior to the study (the a priori sample size) that will be re-estimated during the trial. The proposed re-estimation will be based on results of interim analyses (the re-estimated sample size). This adaptive element in the design of study 2 is motivated by uncertainty in the anticipated effect size and dropout rate. If the actual effect size in the interim analysis is substantially different from the a priori assumed effect size, the a priori estimated sample size may be inadequate to achieve the desired level of statistical power in decision-making. Therefore, interim analysis will be applied to (a) reassess the adequacy of the a priori computed effect size; (b) reassess the initially assumed dropout rate; and (c) replace the a priori sample size by a re-estimated sample size based on these reassessed parameters if needed.

#### A priori sample size

The a priori sample size in study 2 is based on the anticipated change in positive mental health between baseline and one-year treatment follow-up at the clinical setting in the intervention conditions compared to the control group. Compared to arm 3 (i.e., TAU), a medium effect size of Hedges’ g = 0.45 is assumed for both PPI arms followed by a mental health treatment (i.e., arms 1 and 2). The effect size of Hedges’ g = 0.45 is conservatively estimated based on prior studies about PPI effectiveness [21, 45], assuming an overlap in treatment mechanisms when combining a PPI with a subsequent mental health treatment. The Mental Health Continuum – Short Form (MHC-SF) is defined as the primary outcome [46], which is justified in the section on ‘’Data collection’’. Using a pretest-posttest design for this primary outcome, the required sample size was estimated at 207 patients, including 69 patients per treatment arm under a 5% Type I error rate, 80% statistical power, and an equal allocation ratio. While the PPI + mental health treatment arm including loved ones (arm 2) is expected to yield a larger effect size than the PPI + mental health treatment arm without loved ones (arm 1), the actual difference between both effect sizes is unknown due to the novelty of the inclusion of loved ones. In the absence of previous studies, we will conservatively assume identical effect sizes for both arm 1 and arm 2, which can be compared to arm 3 (TAU) with a sufficient degree of certainty. In addition, we still aim to compare arm 1 to arm 2 exploratively, knowing that these specific comparisons may not be adequately powered. Assuming a 35,5% attrition rate between baseline and the 1-year follow up measurement following treatment completion based on previous findings [47, 48], a total sample size of 207/(1–0.355) = 321 participants (*n* = 321) is required, yielding 107 patients per treatment arm. Since the second arm involves one loved one per patient, this arm will contain 214 participants. Thus, a total sample size of 428 participants will be required, including 321 patients and 107 loved ones.

#### Interim analysis: sample size re-estimation

To re-assess the assumed effect size, the sample size and the dropout rate in study 2, incoming data will be monitored by an independent statistician, using an unblinded interim analysis [49]. The sample size reassessment will be conducted when a pre-specified fraction of the data from the originally intended sample are collected, while data collection is ongoing [49]. Although larger data fractions may provide more accurate insights in the expected effect sizes, the current RCT should perform interim analysis relatively early to prevent excessive inclusion of participants. Therefore, an early interim analysis will be performed when 35% of the anticipated complete cases are collected (i.e., when the 6-month post-intervention data of 0.35 * 207 = 72 complete *patient cases* are collected). For the same reason, data from a surrogate timepoint, namely six months following PPI-completion, will be used for interim analysis. While the information obtained may be limited, this selected data fraction is expected to produce relevant information to reassess the effect size estimate (and hence, the sample size) compared to a priori power analysis only [49]. Importantly, this 6-month post-PPI timepoint allows for timely sample size reassessment if the expected average inclusion rate of 5 participants per week is achieved. If the actual average inclusion rate substantially deviates from this assumed inclusion rate of 5 participants per week, a more timely cut-off point for sample size re-assessment will be determined.

The Bayesian approach to sample size reassessment proposed by Wang [50] will be applied to the change score between the pretest and 6 months after intervention completion. Two comparisons will be made, one for each PPI condition (arm 1 and 2) vs. the control group (arm 3). This sample size reassessment method relies on the computation of a Bayesian posterior predictive probability under a non-informative prior distribution to estimate the number of participants (i.e., adjusted sample size) required to draw conclusions, given a pre-specified degree of statistical power and a constant Type I-error rate. In principle, the smallest effect size of the two comparisons will be used for re-estimation of the sample size, but the maximum number of participants to be included is limited at N_max_= 544, including 408 patients and 136 loved ones. This stopping rule is motivated by pragmatic considerations, as this maximum sample size is deemed maximally feasible with the anticipated inclusion rate and the duration of the inclusion phase.

### Study setting

All patients will be recruited from the aforementioned outpatient mental health clinic. This clinic roughly facilitates 120 intakes per month at various sites for adult and elderly care in the province of Limburg, which is situated in the South of the Netherlands. The pre-intake for patients being referred to the outpatient mental health clinic will be facilitated by an eHealth organization providing provisional diagnoses for incoming patients. All patients will learn about study 1 or study 2 during the recruitment procedure, as outlined below.

### Recruitment process

The recruitment procedures below will be applicable to both study 1 and study 2, but differences will be described when necessary. Each patient will undergo a pre-intake at the eHealth organization, which is part of the standard intake procedure for patients being referred to the outpatient mental health clinic. During this pre-intake process, patients will be asked to complete several online questionnaires. Subsequently, each patient will receive a 15-minute consultation with a triage psychologist via telephone. This pre-intake procedure will result in an indication of the patient’s symptoms and a potential diagnosis. Based on this care indication, the outpatient mental health clinic will be advised to either start with treatment or to refer back to the general practitioner. In the latter case, the patient’s symptoms are considered too mild for treatment at the mental health clinic, and participation in the studies will therefore not be possible. Total study eligibility will be assessed using the aforementioned criteria for inclusion and exclusion during this pre-intake. If a patient is deemed eligible for study participation, the triage psychologist will ask whether the patient is interested in receiving study information. A flowchart of the RCT is presented in Fig. 1 below, providing a brief overview of the pre-intake by the eHealth organization, the study enrollment via the O4U-system, the randomization procedure, the key constructs measured in Limesurvey at multiple time points, and the anticipated start of treatment at the outpatient mental health clinic.

If the patient is interested, the patient will receive an email from the eHealth organization, containing a digital link to the Open University research platform (O4U). This O4U-system will be used for managing e-consent and longitudinal measurements, as well as integrating these measurements with another platform responsible for collecting research data (i.e. LimeSurvey). In O4U, patients can read the participant information letter and are asked to provide their e-mail address, which will be used 7 days later to inquire about their willingness to participate in the study. start their 8-week waiting period after the pre-measurement, respectively. Lastly, the patients are asked in O4U to forward a digital link to their loved ones containing a new participant information letter. Given the longitudinal nature of the proposed studies, the anticipated end date of participant recruitment is conservatively estimated at June 2029. More detailed information on the recruitment process can be accessed through the Overview of Medical Research in the Netherlands (NL-OMON57370).

### Randomization and blinding in study 2

Participant assignment will always be concealed for all parties in our research, apart from the following exceptions. Participant assignment may only be disclosed to the participants themselves and a limited number of professionals who need to know at specific points in time, including [1] members of the general research team, [2] the patient’s GP, [3] an IT specialist and [4] an independent statistician. While the general research team may require immediate knowledge of participant assignment via an IT specialist to facilitate all recruitment procedures (e.g., creating the individual eHealth PPI-accounts), the independent statistician may need access to data on group assignment after the 6-month post-PPI measurement to conduct an unblinded and objective interim analysis [51]. During the waiting period, the patient’s GP may be informed about the patient’s study participation. Following randomization, all participants will know whether or not they will receive the eHealth PPI (with or without loved ones). Hence, bias may manifest in terms of treatment expectations [52]. Therefore, data on treatment expectations among participants will be collected and analyzed.

### eHealth positive psychology intervention (eHealth PPI)

An existing online positive psychology intervention (eHealth PPI), which has been successfully implemented for patients with chronic pain [22], will be adapted for patients with various mental health complaints and their loved ones. The current eHealth PPI consists of 9 modules. This intervention aims to increase five protective factors, including self-compassion, positive focus, savoring, gratitude and optimism, which will be offered during an eight-week period. Each participant will be asked to spend approximately 4 h per week on each module. Module 1 presents an introduction about the eHealth PPI, including information on its applicability. Module 1 and module 2 be accessible at the same time. Both module 2 and 3 target self-compassion, which refers to fully accepting oneself, the ability to treat oneself with kindness rather than self-criticism, and recognizing that everyone experiences failures [53]. Both modules aim to teach patients to become more self-reliant when dealing with the emotional consequences of their condition or stressful events. Higher rates of self-compassion are generally associated with improved psychological and physical well-being [54]. Module 4 requires patients to practice the ‘three good things’ exercise on a daily basis [55]. In this module, patients write down three good things that happened that day and why they happened. This exercise aims to shift the focus from a negative orientation towards a more positive one, by raising awareness for the good things in life [56]. Module 5 and 6 are about savoring, which is the capacity to attend, appreciate and enhance the positive experiences in life [57]. From a psychological perspective, savoring is considered a meaningful resource for coping with adversity and amplifying subjective well-being [58]. These modules contain savoring techniques promoting the frequency and intensity of positive experiences in daily life. Module 7 is about gratitude, which represents the ability to identify and acknowledge the valuable things in life [59]. Practicing gratitude frequently may increase well-being [60]. Module 8 contains the Best Possible Self exercise [61], which is aimed at increasing optimism: the tendency to have constructive and positive future expectancies [62]. Optimism has been associated with beneficial coping strategies, applying different coping strategies more flexibly, increased subjective well-being, longevity, and decreased illness [63]. Finally, module 9 aims to prevent relapse, using a personalized maintenance plan. Developing a personalized maintenance plan for positive psychology exercises may encourage future application of the learned methods [64]. During the eHealth PPI, a psychologist from the Open University will organize weekly contact moments (via e-mail) to offer support, to enhance adherence, and to prevent drop-out.

#### Specific design adjustments

The proposed eHealth PPI will address differences in digital skills, low literacy and limited proficiency in the Dutch language within the design of the eHealth PPI. These barriers will be addressed in the eHealth PPI by making the following adjustments: creating short texts, avoiding diagnosis-specific terms, adding or removing theories, optimizing the readability in accordance with Dutch guidelines for addressing low literacy, using persuasive techniques (i.e., newly developed videos, animations, visual content and spoken texts) to optimize engagement, visual attractiveness and attention span. To avoid overwhelming users, explanatory texts and assignments will be positioned in a drop-down menu for users desiring in-depth information about a module.

## Data collection

Implementation procedures of the eHealth PPI will be assessed from a user-perspective in study 1, focusing on: acceptability, feasibility, engagement, adherence, and accessibility. In study 2, outcome variables for assessing clinical effectiveness and procedural implementation will be collected. In addition, study 2 will employ a cost-effectiveness analysis (CEA) and cost-utility analysis (CUA) of the eHealth PPI from a societal perspective, following the recent guidelines issued by Zorginstituut Nederland (ZIN) [65, 66]. In the CEA, the costs and clinical effects of the two eHealth PPI conditions (arm 1 and arm 2) will be weighed against those of the TAU condition (arm 3). Within the CEA, the primary clinical outcome to be collected is a measure for positive mental health. Similarly, in the CUA, the costs and utility outcomes of the two eHealth PPI conditions (arm 1 and arm 2) will be evaluated against those of the TAU condition (arm 3). Within the CUA, the primary outcome to be collected is quality-adjusted life years (QALYs).

### Clinical effectiveness

#### Primary clinical outcome

In positive psychology, positive mental health refers to the presence of human strengths, including a state of emotional, psychological, and social well-being, which empowers individuals to embrace life and build resilience in challenging conditions [41]. Positive mental health will be measured among patients and their loved ones in both studies using the Mental Health Continuum Short Form (MHC-SF). The MHC-SF is used to assess positive mental health in general population studies using 14 items across 3 subscales. The subscales are emotional wellbeing (3 items), social wellbeing (5 items) and psychological wellbeing (6 items). The items reflect the frequency of several feelings in the past month, which are rated ordinally using a 6-point Likert scale: never, once or twice a month, about once a week, two or three times a week, almost every day, and every day. The Dutch version of the MHC-SF has shown a high degree of validity and reliability in the general adult Dutch population. The reliability of the MHC-SF was characterized by a Cronbach’s alpha of 0.89 for the total scale, 0.74 for the subscale of social wellbeing and 0.83 for the subscales of both emotional wellbeing and psychological wellbeing [46]. Due to the small sample size of study 1, the MHC-SF in this study will only be used for measuring preliminary effects within individuals without aiming to guide study adjustments or to establish clinical effectiveness. In study 2, however, the MHC-SF is defined as the primary outcome for clinical effectiveness.

#### Secondary clinical outcomes

In both studies, patients and their loved ones will complete questionnaires designed to measure secondary outcomes within positive psychology, including self-compassion (Self-Compassion Scale – Short Form; SCS-SF) [67], savouring (Savouring Beliefs Inventory; SBI) [68], optimism (Life Orientation Test – Revised; LOT-R) [69], gratitude (Dutch gratitude questionnaire; GQ6) [70], and resilience (Brief Resilience Scale; BRS-NL) [71]. Additional secondary outcomes will be measured among patients and their loved ones, including physical and psychological symptoms (Brief Symptom Inventory; BSI) [72], depression (Patient Health Questionnaire; PHQ-9) [73], and relational satisfaction (Relational Satisfaction Scale; RSS) [74]. All questionnaires are measured ordinally using different Likert scales. Since their applicability extends beyond mental healthcare, these questionnaires have been used in the general Dutch (working) population and adolescent/adult mental health populations. Due to the small sample size of study 1, the SCS-SF, SBI, LOT-R, GQ-6, BRS-NL, BSI, PHQ-9, and RSS in this study will only be used for measuring preliminary effects within individuals without aiming to guide study adjustments or to establish clinical effectiveness. In study 2, however, these questionnaires are all defined as secondary clinical outcomes in study 2.

SCS-SF: the SCS-SF is a 12-item questionnaire measuring six subscales of self-compassion: self-kindness, self-judgment, common humanity, isolation, mindfulness, and over-identification. Each subscale contains two items. To measure each item, the SCS-SF employs an ordinal 5-point Likert scale, ranging from ‘’almost never’’ to ‘’almost always’’. Since Cronbach’s alpha was 0.86, the scale was considered consistent and reliable [67].

SBI: The SBI is a 24-item questionnaire measuring beliefs about savouring (i.e., the ability to appreciate and enhance everyday life), using three 8-item subscales: anticipating pleasure, present moment pleasure, and reminiscing pleasure. Each item is measured ordinally using a 7-point Likert scale, ranging from ‘’strongly disagree’’ to ‘’strongly agree’’. Although a Dutch adaptation is currently not validated, a French version of the SBI was considered internally consistent. Within this SBI version, the three subscales of anticipating pleasure, present moment pleasure, and reminiscing pleasure were characterized by a favourable McDonald’s Omega coefficients of 0.88, 0.86, and 0.85, respectively [75].

LOT-R: the LOT-R is a 10-item questionnaire measuring dispositional optimistic tendencies (i.e., the general tendency to expect positive future outcomes) in two 3-item subscales: optimism and pessimism. The remaining four 4 items are considered fillers. Using a same-day recall period, the LOT-R employs an ordinal 5-point Likert scale to measure all items, ranging from ‘’strongly disagree’’ to ‘’strongly agree’’. A Dutch version of the LOT-R was considered sufficiently internally consistent, based on intraclass correlation coefficients (test-retest reliability) of 0.74 and 0.67 for the subscales of optimism and pessimism, respectively [76].

GQ-6: Apart from measuring optimistic tendencies, the GQ-6 will be used to measure 6 items on gratitude in everyday life using a current-day recall period. Subscales are absent. All items in the GQ-6 are measured ordinally using a 7-point Likert scale, ranging from ‘’strongly disagree’’ to ‘’strongly agree’’. Based on a McDonald’s Omega of 0.75, a Dutch adaptation of the GQ-6 has demonstrated acceptable internal consistency [70].

BRS-NL: the BRS-NL is a Dutch 6-item questionnaire reflecting general resilience traits, using a present-day recall period. The BRS-NL does not contain subscales. Items are measured ordinally using a 5-point Likert scale, ranging from ‘’strongly disagree’’ to ‘’strongly agree’’. Since a Cronbach’s alpha of 0.74 was determined, the BRS-NL has shown acceptable internal consistency [71].

BSI: The BSI is a 53-item questionnaire measuring self-reported psychological and physical symptoms in adult and adolescent mental health populations in Dutch clinical practice, such as grief, anxiety, depression and headaches. Using a 7-day recall period, the BSI measures 9 subscales: somatization, cognitive complaints, interpersonal sensitivity, depression, anxiety, hostility, phobias, paranoid thoughts, and psychoticism. All items are rated ordinally on a 5-point Likert scale, ranging from ‘’not at all’’ to ‘’extremely’’. With Cronbach’s alpha ranging between 0.76 and 0.97 for the 9 subscales, a Dutch version of the BSI was considered internally consistent [72].

PHQ-9: Next, the PHQ-9 is a 9-item questionnaire without subscales. It is primarily used to screen for depressive symptoms in adult and elderly populations suffering from depressive symptoms due to chronic physical illnesses. Using a two-week recall period, the items are measured ordinally on a 4-point Likert scale, ranging from ‘’no’’ to ‘’almost every day’’. With an internal consistency of 0.88 and test-retest reliability of 0.94, a Dutch version of the PHQ-9 was considered highly reliable in a general Dutch primary care sample [77].

RSS: Finally, the RSS is a 12-item questionnaire on certain domains regarding the perceived quality of human relationships, without subscales. This may include intimate relationships, as well as work related relationships, family ties and friendships. This questionnaire was initially applied in the general adult UK population. Using a same-day recall period, all items are measured ordinally on a 6-point Likert scale, ranging from ‘’very dissatisfied’’ to ‘’very satisfied’’. The original version of the RSS was internally consistent with a Cronbach’s alpha of 0.89, indicating strong reliability [74].

#### Collecting background variables for longitudinal change trajectories

In study 2 (RCT), change trajectories within subpopulations of the research sample and between the RCT arms will be modelled over a prolonged period of time. To prepare this modelling process, data on background variables needs to be collected. Based on theoretical considerations, background data on certain demographics (i.e., education and literacy, employment status, sex, and age) and clinical profiles (i.e., severity of psychological complaints, and diagnosis) will be used. Data on these demographic characteristics will be obtained using a separate question about each characteristic. Lastly, data on clinical profiles will be gathered using the aforementioned BSI questionnaire and a separate question on self-reported diagnosis.

### Economic evaluation

#### The application of previously described clinical outcomes for the CEA

In the CEA of study 2, certain outcome variables for assessing clinical effectiveness will also be used to evaluate cost-effectiveness. In the CEA, the aforementioned measure for positive mental health (MHC-SF) will be defined again as the primary outcome. Secondary outcomes will include all scores obtained from the previously mentioned BSI and BRS-NL.

#### Measuring quality adjusted life years for the CUA

In the CUA of study 2, new outcome measures will be collected, including scores on the EuroQol 5-Dimension 5-Level questionnaire (EQ-5D-5L) [78, 79]. The EQ-5D-5L will be used to derive Quality Adjusted Life Years (QALYs), which is defined as the primary outcome in the CUA. The EQ-5D-5L questionnaire is primarily used to assess quality of life in either the general population or subpopulations facing debilitating illness. Given the absence of subscales, the EQ-5D-5L includes 5 items encompassing 5 dimensions related to quality of life: mobility, self-care, daily activities, pain/discomfort, and anxiety/depression. These items are measured ordinally using a same-day recall period, with multiple-choice responses ranging from ‘’no problems’’ to ‘’extreme difficulties’’. With Gwet’s Agreement Coefficient ranging from 0.64 to 0.97 for the 5 dimensions, a Dutch version of the EQ-5D-5 L was considered reliable [80]. However, the clinical meaning of EQ-5D-5 L scores in patients facing mental health complaints has been criticized [81].

To address these concerns in the CUA, adjusted utility scores for estimating quality of life will be measured using the ICEpop CAPability measure for Adults (ICECAP-A) [82] with accompanying Dutch ICECAP-A tariffs [83]. The ICECAP-A was primarily developed to measure human capabilities for generating mental and physical wellbeing in the general adult UK population, using a same-day recall period. Five dimensions (items) of human capabilities are rated: stability, attainment, autonomy, achievement, and enjoyment. All capability items are rated, using an ordinal 4-point scale ranging from low to high. A Dutch version of the ICECAP exists as well, which is characterized by an intraclass correlation of.79. and thus adequate test-retest reliability [84]. In addition, the mental health quality-of-life questionnaire (MHQoL) will be used [85] with accompanying Dutch MHQoL tariffs [86]. Contrary to the generic EQ-5D-5L, the MHQoL aims to specifically measure quality of life in people with mental health issues. Using a same-day recall period, the MHQoL employs a visual analog scale for numerically rating psychological wellbeing and the following 7 dimensions (items) for rating psychological quality of life: self-image, independence, mood, relationships, daily activities, physical health, and the future. In particular, these 7 items are measured ordinally, using 4 response levels ranging from ‘’very satisfied’’ to ‘’very dissatisfied’’. With a Cronbach’s alpha of 0.85, a Dutch version of the MHQoL was considered internally consistent and reliable [85].

#### Measuring societal costs for the CEA and CUA

In both the CEA and CUA, costs will be measured among patients from a societal perspective, including healthcare costs (i.e., health services use and medications), productivity losses (i.e., absenteeism and presenteeism), and intervention costs (i.e., the costs of implementing the intervention). To measure healthcare costs and productivity losses, a 3-month recall period will be used. Consumption of health services, medications, and productivity losses in patients facing psychological complaints will be measured, using the Treatment Inventory of Costs in Patients with psychiatric disorders (TIC-P) [87].

By definition, the TIC-P was developed as a generic questionnaire to estimate societal costs across all Dutch adult mental health populations within inpatient and outpatient clinical settings. Using both categorical multiple choice and numerical open ended questions, the TIC-P aims to measure 3 subscales: general demographics (7 items), healthcare consumption (38 items), and productivity losses in both the formal and informal workspace (12 items) [87]. In the absence of subscale estimates (e.g., Cronbach’s alpha) on reliability, Cohen’s Kappa was still considered highly satisfactory for most separate items on healthcare consumption, and satisfactory for most items on productivity losses [88]. Productivity losses in the workspace are based on absenteeism (i.e., not being able to work) and presenteeism (i.e., reduced productivity in the workspace). Informal productivity losses related to unpaid work will also be measured. Patients will report their hours of absence (i.e., absenteeism) and the additional hours they needed to finish work due to health complaints (i.e., presenteeism).

In addition, intervention costs related to the implementation of the eHealth PPI will be prospectively recorded during the trial using a Microsoft Excel sheet. Following the recent guidelines for economic evaluation issued by Zorginstituut Nederland (ZIN) [65], the measured intervention cost categories are: [1] personnel time, [2] training costs, [3] licensing and platform maintenance, [4] development costs, and [5] technical support. If possible, annual invoices from the participating eHealth organization will be used to assess relevant eHealth PPI implementation costs. More specifically, [1] personnel time includes the amount of time spent by healthcare staff to supervise participants during the eHealth PPI (e.g., providing feedback and responding to alerts). This time investment is a variable cost component and will be derived using digital logs on the eHealth platform and self-reported tracking by staff members from the eHealth organization and Open University. If applicable, [2] additional training costs related to good supervision practices among staff members will be documented as fixed costs, including required resources and preparation time. Annual fees related to [3] licensing and platform maintenance will be retrieved from invoices provided by the eHealth organization. If not covered by licensing fees, additional [4] development costs related to the eHealth software will be recorded and treated as fixed costs, based on provider documentation from the eHealth organization. If [5] technical support costs for participants (e.g., IT helpdesk support) are invoiced separately from licensing and service agreements, the frequency and variable costs per user will be measured using these invoices. Lastly, all healthcare, non-healthcare and intervention cost components will eventually be valued, using the most recent costing manual in Dutch healthcare [66].

### Implementation procedures

#### Measuring procedural constructs

The holistic framework for eHealth evaluation proposed by van Gemert-Pijnen, Nijland [89] will be used to inform an evaluation of eHealth PPI implementation procedures, emphasizing context-sensitive evaluation by employing a participatory approach, advanced mixed methods, and timely measurements of process constructs prior to and during the studies. Guided by these framework principles, five procedural constructs will be addressed from a user-perspective: feasibility, accessibility, adherence, engagement, and acceptability. To measure these constructs, the following measurement methods will be used: written stakeholder feedback, the System Usability Scale (SUS), login frequency data, and post-module process questions. Each measurement method will be explained below.

##### Written stakeholder feedback

Prior to implementing both studies, various stakeholders within the participating mental health clinic will be consulted by e-mail. More specifically, at least five patient representatives, one care manager and one mental health professional will provide written feedback on a digital concept version of the eHealth PPI, which will be used to improve the intervention modules prior to performing study 1 and study 2 [90].

##### System Usability Scale (SUS)

While feasibility refers to the perceived usability and functionality of the eHealth PPI, accessibility examines participants’ ease of access to the eHealth PPI, considering technological literacy [91]. The System Usability Scale (SUS) [92], which is a 10-item questionnaire assessing the perceived usability of digital tools using an ordinal 5-point Likert scale ranging from ‘’strongly disagree’’ to ‘’strongly agree’’, will be used to measure feasibility and accessibility in both studies. The SUS was initially developed to examine adult populations interacting with digital tools. With a Cronbach’s alpha of 0.74, a Dutch version of the SUS was considered internally consistent and reliable for usability assessment [93].

##### Login frequency data

To further explore the accessibility of the eHealth PPI, differences in PPI starting time among participants (i.e., the time between providing informed consent and starting the first PPI module) will be measured in both studies using login frequency data from the eHealth platform. Adherence, defined as the extent to which study participants comply with the basic eHealth PPI protocols (i.e., moving through all required tabs within a module and finishing digital homework assignments), will also be measured in both studies using login frequency data. Lastly, the same login frequency data aims to measure engagement in both studies, defined as the depth of interaction between study participants and the modules of the eHealth PPI. In this context, engagement is measured by assessing both the total time spent on each PPI module and the interactions between study participants and the optional drop-down menus within each PPI module, including additional information and homework assignments.

##### Post-module process questions

The overall acceptability of the eHealth PPI will be measured in both studies using [1] a quantitative rating question and [2] a qualitative, open question after each PPI module (i.e., post-module process questions), requesting study participants: [1] to rate their satisfaction regarding each PPI module on a scale from 1 to 10, [2] to explain their rating in their own words, focusing on the content, features and layout of each PPI module. In addition to measuring acceptability, these post-module process questions may also yield quantitative and qualitative data on the other process constructs (i.e., feasibility, accessibility, adherence, engagement) of the entire eHealth PPI.

### Visual overview of data collection in both studies

The aforementioned questionnaires will be used at several points in time during study 1 and study 2. In both studies, the time spent on each measurement will be at most 30 min. An overview of all proposed measurements for patients and loved ones in both studies is presented in Tables 1 and 2.

**Table 1:** All questionnaires for each measurement in study 1 (RSCD) among patients and loved ones

	T0:pre-PPI-measurement	T1: measure following module 1	T2: measure following module 2	T3: measure following module 3	T4: measure following module 4	T5: measure following module 5	T6: measure following module 6	T7: measure following module 7	T8: measure following module 8	T9: measure following module 9
MHC-SF	x *				x *					x *
BSI	x *									x *
SUS										x *
Post-module process questions		x *	x *	x *	x *	x *	x *	x *	x *	x *
Clinical profile	*x*									
Demographics	x *									
Expectations	x *									
PHQ-9	x*									x*
RSS	x *									x *
LOT-R	x *								x *	x *
SCS-SF	x *		x *	x *						x *
SBI	x *					x *	x *			x *
GQ-6	x *							x *		x *
BRS-NL	x *									x *

*x* = patient; *= loved one; *MHC-SF* Mental Health Continuum – Short Form, *BSI* Brief Symptom Inventory,* SUS* System Usability Scale; Post-module process questions = questions related to the performance of each PPI module from a process evaluation perspective; Clinical profile = mental health complaints, symptomatic severity; Demographics = sex, age, income, education; Expectations = participant expectations regarding the eHealth PPI *PHQ-9* Patient Health Questionnaire – 9, *RSS* Relational Satisfaction Scale, *LOT-R* Life Orientation Test – Revised, *SCS-SF* Self-Compassion Scale – Short Form, *SBI* Savoring Beliefs Inventory, GQ-6 = Gratitude Questionnaire – 6; BRS-NL = Brief Resilience Scale – NL

**Table 2:** All questionnaires for each measurement in study 2 (RCT) among patients and loved ones

	T0: Pre-PPI measurement	T1: Post-measure following immediate PPI completion	T2: 3-month measure following PPI completion	T3: 6-month measure following PPI completion	T4: 9 month measure following PPI completion	T5: 12-month measure following PPI completion	T6: 12-month measure following treatment completion
MHC-SF	x *	x *	*x*	*x*	*x*	*x*	*x*
BSI	x *	x *	*x*	*x*	*x*	*x*	*x*
SUS		x *					
Post-module process questions		x *					
Clinical profile	*x*						
Demographics	x *						
Expectations	x *						
PHQ-9	x*	x*					
RSS	x *	x *					
TIC-P	*x*	*x*	*x*	*x*	*x*	*x*	*x*
EQ-5D-5 L	*x*	*x*	*x*	*x*	*x*	*x*	*x*
MHQoL	*x*	*x*	*x*	*x*	*x*	*x*	*x*
ICECAP-A	*x*	*x*	*x*	*x*	*x*	*x*	*x*
LOT-R	x *	x *					
SCS-SF	x *	x *					
SBI	x *	x *					
GQ-6	x *	x *					
BRS-NL	x *	x *	*x*	*x*	*x*	*x*	*x*

*x* = patient; *= loved one; MHC-SF = Mental Health Continuum – Short Form; BSI = Brief Symptom Inventory; SUS = System Usability Scale; Post-module process questions = questions related to the performance of each PPI module from a process evaluation perspective; Clinical profile = mental health complaints, symptomatic severity; Demographics = sex, age, income, education; Expectations = participant expectations regarding the eHealth PPI; PHQ-9 = Patient Health Questionnaire – 9; RSS = Relational Satisfaction Scale; TIC-P = Treatment Inventory of Costs in Patients with psychiatric disorders; EQ-5D-5 L = EuroQol 5-Dimensions 5-Levels; MHQoL = Mental Health Quality of Life; ICECAP-A = ICEpop CAPability measure for Adults; LOT-R = Life Orientation Test – Revised; SCS-SF = Self-Compassion Scale – Short Form; SBI = Savoring Beliefs Inventory; GQ-6 = Gratitude Questionnaire – 6; BRS-NL = Brief Resilience Scale – Netherlands

## Data analysis

All analyses of (cost-)effectiveness will be performed in accordance with the Intention-To-Treat (ITT) principles, meaning that data from all participants will be analysed according to the study condition to which they were initially assigned, regardless of adherence [94]. Assessment of baseline inequalities between study conditions will be performed and followed up, if needed. Due to the longitudinal nature of study 1 and study 2, dropout and other processes causing missing data are expected [95]. Appropriate missing data handling techniques will be applied after assessment of the magnitude, mechanisms, and patterns of missing data [96]. A one-sided alfa of 0.05 will be applied in both studies. Since multiple comparisons between RCT arms will be performed in study 2, this significance level of 0.05 will be adjusted to manage Type I error probability, using the Holm-Bonferroni method [97].

### Clinical effectiveness

#### Primary analyses

The primary clinical analyses aim to examine whether the eHealth PPI has a significant clinical effect on positive mental health (i.e., MHC-SF), which will be evaluated in the RCT of study 2. The primary analysis will be an Analysis Of Covariance (ANCOVA), which aims to compare the three independent RCT conditions on positive mental health scores at the 12-month post-PPI measurement, with the pre-PPI measurement serving as a covariate.

#### Secondary analyses

The secondary clinical analyses of study 2 aim to evaluate whether the eHealth PPI significantly affects other outcome measures, in addition to examining the effects of including loved ones in the eHealth PPI. Outcome scores on the other positive psychology constructs (i.e., SCS-SF, SBI, LOT-R, GQ-6, and BRS-NL), physical and psychological symptoms (i.e., BSI), depression (i.e., PHQ-9), and relational satisfaction (i.e., RSS) will be addressed in both studies. In study 2, an ANCOVA will be used again, employing the pre-PPI-measurement as a covariate. Using this method, a comparison will be made between the pre-PPI measurement and the immediate post-PPI measurement to analyse the scores on the SCS-SF, SBI, LOT-R, GQ-6, BRS-NL, BSI, PHQ-9, and RSS across the three independent RCT conditions. Using this approach, the effects of including loved ones in the second condition *(eHealth PPI for patients and loved ones)* will also be examined, relative to the other conditions. An additional visual analysis — considered foundational for single-case designs [98] — will be employed in study 1 to identify preliminary effects within individuals for descriptive purposes without aiming to establish clinical effectiveness or to guide study adjustments.

In study 2, multilevel regression modelling will be used to examine and compare the average longitudinal trajectory of change on the primary outcome (i.e., MHC-SF) across the three RCT arms. Based on theoretical considerations, certain demographics (i.e., education and literacy, employment status, sex, and age) and clinical profiles (i.e., severity of psychological complaints, and diagnosis) are considered relevant group-level moderators for modelling such change trajectories. Using these moderators in a multilevel regression model, moderation analyses will be employed to examine variations in longitudinal change trajectories between the RCT conditions on the primary outcome (i.e., MHC-SF) and relevant secondary outcomes (i.e., BSI, MHQoL, and BRS-NL) across the seven RCT measurement points. Using the same moderation analyses, the average trajectory of change for certain subpopulations will be examined across the seven RCT measurement points to evaluate how different subpopulations respond to the eHealth PPI over time. Notably, moderation analyses for subpopulations may reveal different associations between group-level moderators and PPI effectiveness, even if these associations cancel each other out in the overall change comparisons between the three RCT conditions [99].

### Economic evaluation

#### Valuation of clinical outcomes

In the CEA of study 2, clinical outcomes derived from the MHC-SF, BSI and BRS-NL will be valued, using specific conversion guidelines [65, 66]. In the CUA of study 2, utility scores derived from the EQ-5D-5 L, MHQoL and ICECAP-A will be converted to QALYs using existing methods, including the area-under-the-curve method [100]. While the EQ-5D-5 L scores will serve as the main quality of life outcomes, the MHQoL and ICECAP-A scores will be used in a sensitivity analysis. In general, MHQoL and ICE-CAP-A scores are expected to reflect wellbeing differently in patients facing psychological complaint, when compared to EQ-5D-5 L scores [81].

#### Valuation of societal costs

The sum of healthcare costs, productivity losses, and intervention costs will be estimated for the eHealth PPI conditions (arm 1 and arm 2) and the TAU condition (arm 3), yielding the societal cost per RCT arm. Firstly, the reported consumption of each health service type and medication in the TIC-P will be multiplied by a respective reference price, using the guidelines for economic evaluation issued by ZIN [65, 66]. Secondly, productivity losses due to absenteeism and presenteeism, as measured by the TIC-P, will also be addressed. Absenteeism will be valued using the friction cost method [100]. Using this method, the reported hours of absence will be multiplied by the average hourly rate of a working individual in The Netherlands, to a maximum of 12 weeks. After 12 weeks, initial productivity losses due to absenteeism are assumed to be restored. Presenteeism will be derived from the additional hours that participants need to finish their work due to health complaints. Likewise, these additional hours are multiplied by the average hourly rate of a working individual in The Netherlands.

Within the eHealth PPI, [1] personnel time will be valued using standard reference prices per hour, based on the staff’s professional category (e.g., psychologist, student psychologist, and IT support). Additional [2] training costs, as measured by resources and preparation time, will be valued. Depending on training prices and the total number of required trainings during the RCT, this cost component will be calculated by multiplying both. In addition, [3] licensing and maintenance costs, [4] development costs, and [5] technical support costs will be monetized, using invoices provided by the eHealth organization. If invoices are issued on a per-user basis, the unit price per participant can be applied directly. However, if invoices are issued as an annual organizational fee, the average annual cost per user will be calculated by dividing the total annual fee by the annual number of eHealth PPI participants (within the billing period).

In the second RCT arm, both patients and their loved ones will have an individual user account to engage with the eHealth PPI. Hence, total intervention costs of this eHealth PPI condition (arm 2) are expected to outweigh those of the patient-only eHealth PPI condition (arm 1). All healthcare, non-healthcare and intervention cost components will be indexed to the most recent base year, using the latest costing manual in Dutch healthcare [66]. If necessary, all estimated costs will be extrapolated to an extended time horizon that is not covered by the 3-month recall period. Following the ITT-principles throughout the entire RCT duration, the total sum of healthcare costs, non-healthcare costs, and intervention costs will be calculated and averaged per participant within each RCT condition, yielding an estimate of the average societal cost per RCT condition. These estimates will then allow for comparative base-case analyses of cost-effectiveness.

#### Cost-effectiveness: base-case analyses

The probability of the two eHealth PPI conditions (arm 1 or arm 2) being cost-effective compared to TAU (arm 3) will be calculated from a societal perspective. Following the Dutch guidelines for economic evaluation in healthcare [65, 66], costs and effects extending beyond a 1-year follow-up period will be adjusted to their present value by an annual discount rate of 3% and 1.5%, respectively. In addition, all costs will be indexed to the most recent base year deemed appropriate. An additional scenario may be considered, assuming that costs and effects within and beyond the 1-year follow-up period should be adjusted to their present value. In addition to performing an ITT-analysis of cost-effectiveness, a complete-case cost-effectiveness analysis will be employed, using only data from participants who completed all measurements. Baseline differences in costs and outcomes between the trial arms will be analyzed and, if necessary, accounted for using regression analysis [101].

In the proposed cost-effectiveness analysis (CEA) and cost-utility analysis (CUA), the incremental cost-effectiveness ratio (ICER) will be calculated to facilitate two pairwise comparisons: (I) arm 1 vs. arm 3, and (II) arm 2 vs. arm 3. For both comparisons, the ICER represents the difference in societal costs between an eHealth PPI condition (arm 1 or arm 2) and the TAU condition (arm 3), divided by the corresponding difference in clinical outcomes (e.g., QALYs and MHC-SF scores). The robustness of the ICERs obtained will be assessed through non-parametric bootstrapping, using 1000 samples. This approach aims to predict the joint distribution of costs and effects within a cost-effectiveness plane (CE-plane) [102].

Subsequently, the cost-effectiveness of the two eHealth PPI conditions compared to TAU depends on the willingness to pay (WTP) for an incremental gain in effectiveness associated with a new treatment [100]. In the Dutch healthcare system, WTP thresholds of €20,000, €50,000 and €80,000 per QALY gained exist, depending on the burden of disease [65, 66]. Based on the Integrated Disease Burden Calculator (IDBC) [103], a WTP of €20,000 per QALY gained may be considered an appropriate threshold. All assumptions for this calculation are provided in Appendix 1. Using a WTP continuum ranging from €0 to €80,000, a cost-effectiveness acceptability curve (CEAC) will be presented, displaying the likelihood that the bootstrapped ICERs of both PPI conditions compared to TAU fall below the assumed WTP threshold of €20,000 per QALY gained. This method reflects the probability that either of the two eHealth PPI conditions (arm 1 or arm 2) is cost-effective compared to TAU (arm 3). Using the same CEAC, the probability of arm 2 being cost-effective compared to arm 1 - and vice versa – will also be computed. To address uncertainties affecting the base-case analyses, the ICERs obtained will be recalculated using sensitivity analyses on the parameters of costs and health service use [104].

#### Budget impact analysis

Using guidelines from the International Society for Pharmacoeconomics and Outcomes Research (ISPOR) [105], a budget impact analysis (BIA) will be performed to examine the financial consequences of implementing the eHealth PPI on a national level. In short, the analyses will be conducted from three stakeholder perspectives: [1] a societal perspective [2], a healthcare perspective, and [3] a healthcare insurer perspective. Within the societal perspective, healthcare costs and non-healthcare costs will be examined. Similar to the base-case analysis, healthcare costs will be estimated, using standard unit prices in Dutch healthcare [106]. For the other perspectives, national Dutch tariffs from Nationale Zorg Autoriteit (NZA) will be applied. For each stakeholder perspective, the following scenarios will be assessed: an eHealth PPI adoption rate of 40%, 60% and 80% among the target population. In addition, an extreme scenario will be modelled, assuming a 100% adoption rate. All scenarios will be compared to a base-case scenario, assuming a 0% adoption rate among the target population.

To explore these scenarios, a health-economic simulation model will be developed based on appropriate assumptions regarding the distributions of costs and effects. Costs will be modelled over the short term (12 months) and long term (36 months). Long-term costs will be discounted. Costs and outcomes will be extrapolated to a time horizon of 3 years for the target population. To address outcome uncertainty, sensitivity analyses with varying cost and discounting parameters will be conducted. To facilitate reproduction of the employed analyses, characteristics of each parameter (e.g., volumes, cost prices) and the model itself will be reported.

### Implementation procedures

#### Quantitative and qualitative analyses

In accordance with the aforementioned framework for eHealth evaluation [89], advanced mixed methods will be used to inform an evaluation of eHealth PPI implementation procedures. In addition to analysing participants’ quantitative post-module ratings using descriptive statistics (e.g., means, frequencies, standard deviations), participants’ qualitative responses to the post-module process questions will be summarized using thematic analysis with coding trees in both studies, until data saturation is reached [107]. In study 2, an Analysis Of Variance (ANOVA) may identify differences in eHealth PPI login frequency data (i.e., adherence and engagement) and SUS outcomes (i.e., feasibility and accessibility) across the RCT arms, while correlation and regression analyses may explore the associations between SUS outcomes, eHealth PPI login frequencies, the primary outcome (i.e., MHC-SF), and secondary outcomes. Data on all aforementioned procedural constructs may potentially explain variations in preliminary intervention effects (study 1) and (cost-)effectiveness outcomes across RCT conditions (study 2). Therefore, triangulation of quantitative and qualitative data will be considered in both studies [108]. Different PPI starting times between patients and loved ones are anticipated in both studies (e.g., due to a delay in obtaining consent from the patients’ loved ones), which may particularly affect SUS outcomes representing the intervention’s accessibility and feasibility. Therefore, average differences in PPI starting time between patients and loved ones (study 1) and across the RCT arms (study 2) will be assessed using an ANOVA. If significant differences in PPI starting time are found, SUS outcomes will be examined using regression modeling, employing the PPI starting time as a covariate.

## Medical-ethical approval and trial registration

Importantly, the two proposed trials (study 1 and study 2) have been approved by the accredited medical-ethical committee of Zuyderland Medical Centre in the Netherlands (NL87751.096.24). In addition, the two proposed studies have been formally registered in the Overview of Medical Research in the Netherlands (NL-OMON57370) since 17 March 2025. If applicable, relevant protocol amendments will be communicated to the respective medical-ethical committee in due time.

### Research ethics

General research ethics will guide the implementation of our studies. From an ethical perspective, all incoming patients will have an opportunity to be assessed for study eligibility during this inclusive recruitment process, regardless of their initial complaints and demographic characteristics. All research activities in the proposed O4U-system adhere to current participant privacy regulations within Dutch mental healthcare. The e-consent file will be established in accordance with the latest regulatory requirements in Dutch research trials involving humans [109], including a mandatory reflection period for participants after receiving study information and before providing consent. In the e-consent file, all patients and their loved ones will be asked to confirm whether they had enough time to decide upon study participation.

Following the General Data Protection Regulation (GDPR), every participant retains the right to withdraw from the proposed studies at any given time. In addition, all participants hold the GDPR-compliant right to be forgotten at any time, meaning they can no longer be contacted for the study or any follow-up research. However, the researchers retain the formal right to use a participant’s anonymized data collected prior to study withdrawal. The related software and platforms used to implement the eHealth PPI and research measurements comply with contemporary patient privacy regulations and procedures regarding (digital) e-consent. Following completion of the waiting period, all patients will receive an official intake for mental health treatment at the mental health clinic, regardless of whether the patients in the PPI conditions have actually completed the eHealth PPI.

Lastly, the proposed sample size re-assessment in the interim analysis of study 2 aims to prevent including too many participants. However, by the time this interim analysis is conducted, there may still be a minor risk of more participants being included than necessary to obtain statistical power. In this case, all excess participants are allowed to complete the eHealth PPI, but will no longer receive unnecessary follow-up measurements. This approach aims to minimize participant burden, while preserving statistical power.

### Managing adverse events

In addition to general research ethics, a plan for managing adverse events (AEs) and serious adverse events (SAEs) has been developed. Following medical-ethical guidelines, SAEs resulting in death or life threatening injury will be reported to the accredited medical-ethical committee of Zuyderland Medical Centre within 7 days of first knowledge. Subsequently, a related report will be completed for the medical-ethical committee within a follow-up period of 8 days. All other SAEs will be reported within a maximum period of 15 days after obtaining knowledge of the events. In addition, a distinction will be made between SAEs that are likely due to the intervention and SAEs that presumably stem from other causes. An annual safety report about all AEs during the study trials will be provided, including the SAEs. To distinguish between general AEs and different types of SAEs, the research team will adhere to the protocols of the respective medical-ethical committee. In addition, three members of the Open University (OU) research team have completed the latest Dutch course on Good Clinical Practices (GCP) in May 2025, containing valuable information about managing AEs and SAEs in clinical research. In line with the medical-ethical guidelines, both general liability insurance and participant insurance will be provided to financially compensate those who may suffer from trial participation. Following careful deliberation with the accredited medical-ethical committee of Zuyderland Medical Centre, a separate Data Safety Monitoring Board (DSMB) was not required.

During the waiting period, a patient’s health status may deteriorate as a result of symptomatic aggravation, which may or may not become an adverse event [9]. Using a safety plan and a disclaimer in the eHealth PPI, patients will be informed on how to reach out to their local GP or urgent care GP. Participants are asked to fill out this safety plan during the first week of participation. In this safety plan and in each intervention module, the following disclaimer will be presented: *‘’Support during this study will primarily be offered through email contact. However*,* your emails may not be answered on a daily basis. Therefore*,* this form of communication is insufficient in urgent cases. In case of an emergency*,* you should always contact your general practitioner.’’* Potential side effects and clinical benefits will be explained to patients and their loved ones, using a comprehensive participant information letter. In severe cases, included patients may be hospitalized in an inpatient mental health clinic. While hospitalized patients are ethically allowed to continue using the eHealth PPI, many of them will likely drop out.

## Discussion

A study protocol aimed at examining a transdiagnostic eHealth PPI has been developed, using two studies. Study 1 (RSCD) and study 2 (RCT) aim to examine the clinical effectiveness, cost-effectiveness and implementation procedures of an eHealth PPI for patients awaiting mental health treatment and their loved ones. The proposed eHealth PPI will address transdiagnostic factors, meaning that the underlying PPI treatment principles are generally applicable across various mental health conditions, regardless of diagnosis. Several strengths can be identified. Firstly, to our knowledge, this is the first paper aimed at evaluating a threefold objective in the context of eHealth PPIs for both patients and their loved ones. Secondly, the inclusion of loved ones in the eHealth PPI is considered an empirically supported innovation, which could enhance the intervention’s effects among both patients and their loved ones. Thirdly, both studies will be conducted within a single outpatient mental health clinic containing multiple treatment sites in the Dutch province of Limburg, supervised by a unified management team. As a result, the implementation process of the eHealth PPI can be organized relatively efficiently across the different treatment sites. Fourthly, the process of developing a transdiagnostic eHealth PPI is deemed highly feasible, since the content of the modules has already been applied successfully in multiple patient populations, including patients with depression, anxiety and chronic pain [21–23]. Fifthly, an experienced e-health organization and IT department of the Dutch Open University will facilitate the implementation process of the proposed eHealth PPI. Since this intervention will be entirely integrated into existing organizational layers of the participating mental health clinic, we do not expect our project to be highly burdensome for clinicians. In fact, the current project clearly aligns with contemporary developments in the field of hybrid mental healthcare [1, 110]. Lastly, considering the includable range of patient populations, we aim to examine which patients are most likely to benefit from the eHealth PPI, focusing on both intervention group comparisons and on trajectories of smaller subpopulations. This approach may facilitate the development of personalized eHealth protocols aimed at enhancing individual patient outcomes in future research.

Apart from the anticipated strengths, certain operational challenges in performing the studies need to be addressed. Firstly, not all patients are equally knowledgeable about eHealth, neither are they equally literate. In fact, intervention adherence among participants may be compromised due to low eHealth literacy [111]. To address this challenge, an evaluation of implementation procedures will be conducted to examine and optimize the eHealth PPI from a user-perspective, using a variety of quantitative and qualitative approaches prior to and during both studies. Secondly, dropout among patients and loved ones may be related to the weekly 4-hour time investments required to complete an intervention module, the extensive measurements and confrontational questionnaires [112]. To mitigate this risk, precautionary measures will be taken to ensure that the time spent on each measurement will be at most 30 min. These measures include using brief, yet validated questionnaires addressing the research objectives. Thirdly, the two eHealth PPI arms (with and without loved ones) will be compared to each other exploratively in study 2, since there is no information about effect sizes available to perform a fully powered analysis. Until all data are collected, it remains unclear whether the sample size of study 2 is adequate for this purpose. In fact, the expected difference in effects between both PPI arms is likely smaller than the difference between each PPI arm and the TAU arm, which was considered the foundation for the a priori sample size calculation. If this turns out to be the case, the statistical power may fall below the conventional 80% threshold and true differences may remain undetected. In that case, any conclusions about the generalizability of upcoming findings should be drawn with greater caution. That being said, the interim analyses aim to estimate the actual effect size, which is informative for both PPI forms (with and without loved ones) in our research setting. More specifically, this estimation of the actual effect size may guide future research initiatives aimed at developing a confirmative study design employing both PPI forms with a sufficient degree of statistical power, without compromising the most important contrasts (i.e., PPI for patients vs. TAU and PPI for patients + loved ones vs. TAU). Fourthly, assessing (cost-)effectiveness may be complicated due to the heterogeneity of the target populations addressed in the current project. Apart from loved ones, various clinical patient populations are eligible for participation. Since clinical factors may substantially affect costs and treatment outcomes, certain clinical profiles (i.e., severity of psychological complaints, and diagnosis) will be included as covariates in both studies. Fifthly, the proposed recruitment procedures at the Dutch mental health clinic and the eHealth organization are aimed at minimizing selection bias, which may optimize the representativeness of vulnerable patient populations in our studies. However, a selectively induced bias among participants may not be ruled out entirely, since vulnerable target groups (e.g., individuals with low literacy) are less likely to seek psychological treatment and to participate in digital research [113, 114]. Lastly, the southern regions in the Dutch province of Limburg are characterized by significant socio-economic challenges [113], which could affect the broader generalizability of upcoming study findings on a national level in The Netherlands.

Over the next 4 years, interim (cost-)effectiveness analyses will be performed to carefully monitor the preliminary results, while data collection is still ongoing. Definitive conclusions regarding (cost-)effectiveness will be drawn when the data collection is fully completed, which is expected to take 5–6 years. In addition to supporting loved ones, the proposed eHealth PPI may support the patient’s mental health relatively early during the waiting period, potentially reducing the total number of subsequent mental health treatment sessions required for clinical remission. In that case, the proposed eHealth PPI might indirectly improve the availability of mental health professionals, thereby reducing the long-term burden of waiting lists in mental healthcare and potentially making it a (cost-)effective intervention. Addressing the proposed research gaps may enhance our academic understanding of transdiagnostic eHealth PPIs, including their clinical and financial implications. Hence, upcoming study results may be used to inform resource allocation decisions regarding the implementation of eHealth PPIs within Dutch mental healthcare and potentially beyond.

## Supplementary Information

Supplementary Material 1

## Data Availability

No datasets were generated or analysed during the current study.

## Appendix

WTP-threshold analysis using the Integrated Disease Burden Calculator (IDBC)

### Objective

This appendix aims to quantify an ethically justified estimate of the WTP-threshold associated with the disease burden of a transdiagnostic (i.e., heterogeneous) mental health population using the Integrated Disease Burden Calculator (IDBC) (103).

### Methodology

The study population consists of adults aged 18 and older. All mental health disorders are included, apart from psychotic disorders and acute suicidality. Using a recent systematic review of 37 studies on internet interventions for various mental health populations (37), the following assumptions were made:

**Table:**

Assumptions	Value	Interpretation
Mean age	40,2 years	Typical age of eHealth participants
Percentage of males	37%	Men are generally underrepresented in mental health research

In addition, the following assumptions were made:

**Table:**

Assumptions	Value	Interpretation
QALY discount rate	1,5%	Standard tariff in economic evaluation
Standard error surrounding QALYs	1.0%	Neutral - unknown
Remaining QALYs with standard care	20,5 QALYs	Assumed utility of 0,65 on average for all mental health disorders combined, apart from psychotic disorders and acute suicidality. and a discounted life

### Outputs

Using the aforementioned assumptions, the following IDBC outputs were generated:

**Table:**

Outcome	Value	Interpretation
Remaining QALYs with standard care	20,5 QALYs	Average functioning under treatment as usual
QALYs without the diseases	27,52 QALYs	Healthy life expectancy at age 40 (averaged for men and women)
Proportional shortfall	0.26 (95%-confidence interval: 0.17–0.32)	26% of healthy life years lost due to mental illnesses; 95% confidence interval ranges between 0.17 and 0.32
Probability of exceeding a proportional shortfall of 0.4	0%	The entire distribution is below the proportional shortfall of 0.4

### Proportional shortfall – recommendations regarding the WTP threshold

**Table:**

Proportional shortfall	Recommended WTP threshold	Interpretation
< 0.4	€20.000 per QALY gained	Relatively low disease burden
0.4–0.85	€50.000 per QALY gained	Moderate disease burden
> 0.85	€80.000 per QALY gained	Relatively high disease burden

Since the proportional shortfall is estimated at 0.26 (95%-confidence interval: 0.17–0.32), the transdiagnostic mental health population falls entirely below the 0.4 proportional shortfall threshold under the IDBC framework. In the absence of probabilistic justifications for higher thresholds, a WTP of €20.000 per QALY gained is currently considered the most appropriate threshold for assessing the cost-effectiveness of the proposed eHealth PPI.

### Contextual limitations

Although the proposed calculation under the IDBC model provides a robust estimate of health losses on the individual level, it does not seem to capture societal constraints, including systemically prolonged waiting times in Dutch mental healthcare. Unmet needs and delayed access to treatment (due to waiting lists) are not visibly reflected in QALY-based shortfall estimates. In addition, the model does not account for treatment scarcity and equity considerations. Lastly, the model currently relies on rather uncertain average utilities, which may underestimate the burden of disease for overrepresented subgroups with more severe conditions within the heterogeneous mental health population. While a WTP threshold of €20.000 per QALY gained is currently justified from a strict IDBC perspective, other societal, ethical and system-level considerations may enable a more generous threshold for assessing the cost-effectiveness of the proposed eHealth PPI.

## Abbreviations

AE: Adverse Event
ANCOVA: Analysis Of Covariance
ANOVA: Analysis Of Variance
BIA: Budget Impact Analysis
BRS-NL: Brief Resilience Scale – Netherlands
BSI: Brief Symptom Inventory
CEA: Cost-Effectiveness Analysis
CEAC: Cost-Effectiveness Acceptability Curve
CE-plane: Cost-Effectiveness Plane
CUA: Cost-Utility Analysis
DSMB: Data Safety Monitoring Board
EQ-5D-5L: EuroQol 5-Dimensions 5-Levels
GCP: Good Clinical Practices
GDPR: General Data Protection Regulation
GP: General Practitioner
GP-MHP: General Practice Mental Health Professional
GQ-6: Gratitude Questionnaire − 6
ICECAP-A: ICEpop CAPability measure for Adults
ICER: Incremental Cost-Effectiveness Ratio
IDBC: Integrated Disease Burden Calculator
ISPOR: International Society for Pharmacoeconomics and Outcomes Research
ITT: Intention-To-Treat
LOT-R: Life Orientation Test - Revised
MHC-SF: Mental Health Continuum – Short Form
MHQoL: Mental Health Quality of Life
Nmax−total: Maximum number of participants to be included
NSSR−total: Re-assessed number of participants to be included
NZA: Nationale Zorg Autoriteit (National Dutch Care Authority)
OU: Open University (of the Netherlands)
O4U: Open University Research Platform
PHQ-9: Patient Health Questionnaire − 9
PPI: Positive Psychology Intervention
QALY: Quality-Adjusted Life Year
RCT: Randomized Controlled Trial
RSCD: Replicated Single-Case Design
RSS: Relational Satisfaction Scale
SAE: Serious Adverse Event
SBI: Savoring Beliefs Inventory
SCS-SF: Self-Compassion Scale – Short Form
SUS: System Usability Scale
TAU: Treatment As Usual
TIC-P: Treatment Inventory of Costs in Patients with psychiatric disorders
WTP: Willingness To Pay
ZIN: Zorginstituut Nederland (Dutch Care Institute)
